# Evaluating Cardiovascular Disease Risk in Male Forensic Inpatients Using QRISK-3: A Quality Improvement Project

**DOI:** 10.1192/bjo.2026.11488

**Published:** 2026-06-30

**Authors:** Elif Sogut

**Affiliations:** Brockfield House, Wickford, United Kingdom

## Abstract

**Aims:**

People with severe mental illness have a life expectancy that is 10 to 20 years shorter than the general population. Cardiovascular disease plays a major role in this gap. Additionally, forensic inpatient population often experiences prolonged admissions and has limited access to community physical health services. QRISK3 is a tool that estimates an individual's 10-year risk of having a heart attack or stroke. This project aimed to assessQRISK3 scores in forensic low and medium-secure male wards and offer atorvastatin for primary prevention when appropriate.

**Methods:**

A snapshot review was conducted in November 2025 among 59 inpatients in Brockfield House low and medium secure male wards. 5 patients younger than 25 years old were excluded as QRISK tool is only valid for patients aged 25–84 years. QRISK3 scores were calculated for all eligible individuals using Mobius (an electronic record system) and patient interviews. Patients with a QRISK3 score of 10% or higher were informed of their increased cardiovascular risk and offered atorvastatin based on primary prevention guidelines (https://cks.nice.org.uk/topics/cvd-risk-assessment-management).The collected data was saved in a password-protected Excelspreadsheet.

**Results:**

A total of 54 patients were included in the analysis. The mean age was 41.98 years (range 25–69).14% of patients were current smokers (n=8), 50% were ex-smokers (n=27), 31% had diabetes mellitus (n=17), and 11% were prescribed antihypertensive medication (n=6). The mean BMI was 32.33 kg/m², 91% of patients had a BMI ≥25 kg/m² (n=49), indicating overweight or obesity. 16 patients had a QRISK3 score ≥10%. Of these, 11 patients were already prescribed a statin. The remaining 5 patients were offered atorvastatin for primary prevention; 4 agreed to commence treatment following consultation, while one declined. QRISK3 scores were documented in Care Programme Approach (CPA) reports for all patients in whom they were calculated. On average, it took 12 minutes and 36 seconds to gather information and calculate the QRISK3 score.

**Conclusion:**

This QIP demonstrates that cardiovascular risk assessment using QRISK3 in forensic psychiatric inpatient settings is practical, time-efficient, and clinically valuable.Integrating QRISK3 scoring into routine CPA documentation may improve identification of patients at increased cardiovascular risk and increase appropriate statin prescribing for primary prevention in line with NICE guidelines.

